# Cellular Angiofibroma of the Perineal Region With Tortuous Vessels: A Report of Three Cases

**DOI:** 10.7759/cureus.80969

**Published:** 2025-03-21

**Authors:** Takahiro Seki, Yoichi Kaneuchi, Michiyuki Hakozaki, Toru Hirai, Shoki Yamada, Osamu Hasegawa, Takuya Nikaido, Yoshihiro Matsumoto

**Affiliations:** 1 Department of Orthopaedic Surgery, Fukushima Medical University School of Medicine, Fukushima, JPN; 2 Higashi-Shirakawa Orthopaedic Academy, Fukushima Medical University School of Medicine, Fukushima, JPN; 3 Department of Orthopaedic Surgery, Jusendo General Hospital, Fukushima, JPN; 4 Department of Diagnostic Pathology, Fukushima Medical University School of Medicine, Fukushima, JPN; 5 Department of Radiology and Nuclear Medicine, Fukushima Medical University School of Medicine, Fukushima, JPN

**Keywords:** cellular angiofibroma, mri, soft tissue tumor, tortuous vessel, vascular proliferation

## Abstract

Cellular angiofibroma (CA) is a slow-growing, painless benign soft tissue tumor that commonly arises in the superficial soft tissue of the vulvovaginal or inguinoscrotal regions. Since CA shares many pathological and imaging similarities with angiomyofibroblastoma and aggressive angiomyxoma, its preoperative imaging diagnosis can be challenging. To the best of our knowledge, this is the first report describing tortuous vessels around the tumor as imaging findings to assist in diagnosing CA and distinguishing it from angiomyofibroblastoma and aggressive angiomyxoma in the perineal region.

## Introduction

Cellular angiofibroma (CA) commonly occurs in adults [1]. Male and female individuals are affected in almost equal numbers, with a peak incidence in their seventh decade of life in men and fifth decade in women [1]. Angiomyofibroblastoma (AMF) and aggressive angiomyxoma (AA) are also benign soft tissue tumors around the perineal regions. However, these three types of tumors share similarities in imaging findings of magnetic resonance imaging (MRI) and immunohistochemistry. Therefore, the accurate preoperative diagnosis is challenging. We herein report the cases of three patients in which tortuous vessels around the tumor helped diagnose CA on MRI.

## Case presentation

Case 1

**Figure 1:**
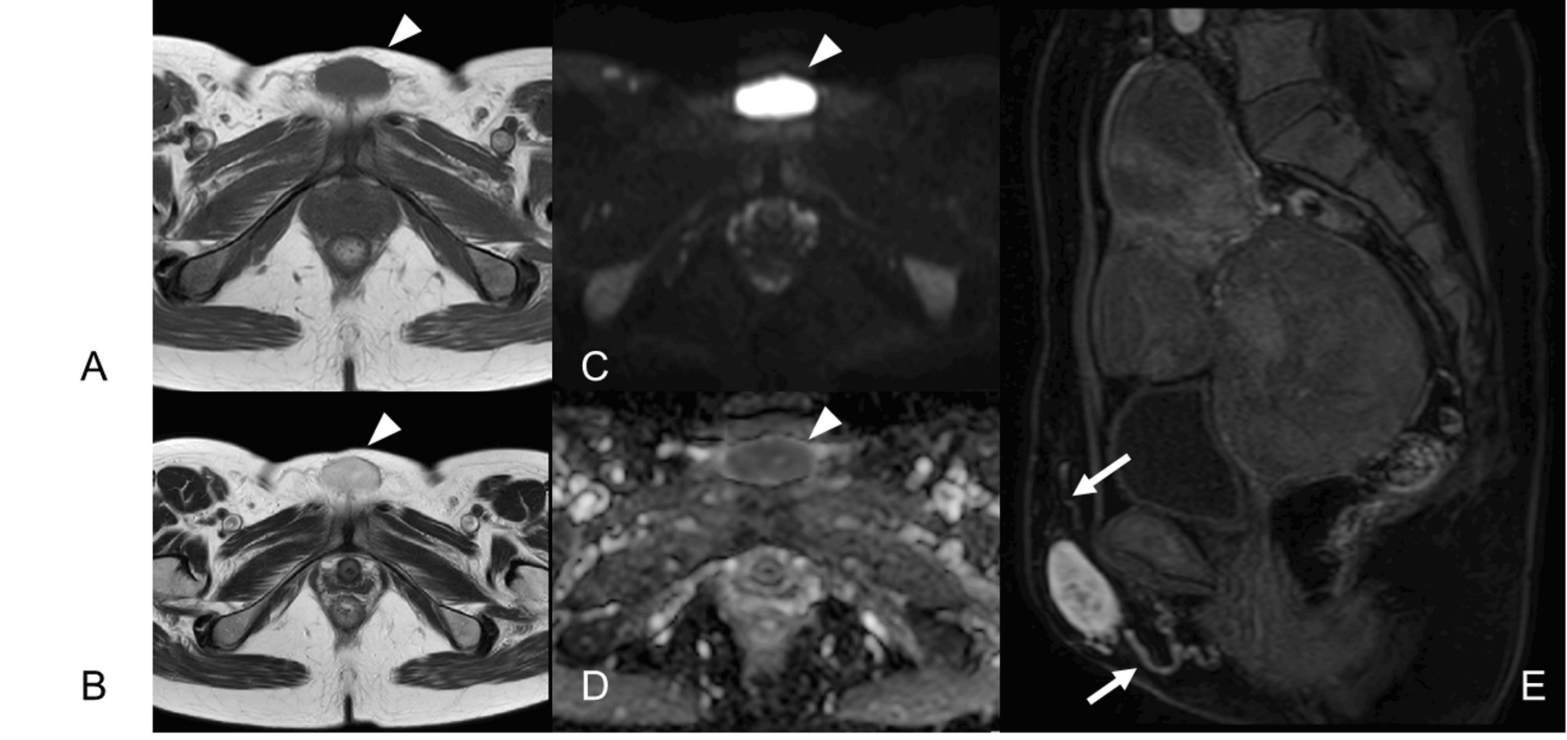
MRI of the pelvis (Case 1) A well-defined 3.9×3.9×2.0 cm tumor (arrowheads) is present within the subcutaneous fat tissue, showing iso-intensity on T1-weighted image (A: axial view) and high-intensity with a low-intensity capsule on T2-weighted image (B: axial view), high-intensity on DWI (C: axial view), and low-intensity on ADC (D: axial view). The tumor showed almost homogenous enhancement with gadolinium on T1-weighted fat suppression, and tortuous vascular structures (arrows) were proliferating around the tumor (E: sagittal view). DWI: diffusion-weighted imaging

A 54-year-old previously healthy Japanese female patient was referred to our hospital with a one-month history of a gradually growing soft tissue mass in the perineal region. On physical examination, a mobile, elastic, soft mass with a diameter of 4 cm was observed on the pubic surface, without tenderness or redness. MRI revealed the well-defined tumor exhibiting iso-intensity on T1-weighted imaging (T1-WI) (Figure 1A), high intensity on T2-weighted imaging (T2-WI) (Figure 1B), high-intensity on diffusion-weighted imaging (DWI) (Figure 1C), low-intensity on apparent diffusion coefficient (ADC) (Figure 1D), and relatively homogenous enhancement on the gadolinium-enhanced T1-WI fat-suppression image; tortuous vessels surrounding the tumor were also detected (Figure 1E).

**Figure 2:**
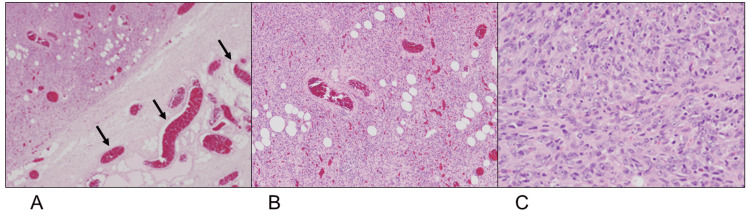
Histopathological examination, hematoxylin & eosin staining (Case 1) A proliferation of blood vessels (arrows) was observed at the tumor periphery (A: magnification, ×12.5). Spindle cells with short spindle-shaped nuclei proliferated densely within the tumor, which contained various-sized blood vessels (B: magnification, ×40). The stroma was mildly edematous with lymphocytic infiltration (C:  magnification,×400).

Based on the needle biopsy results, the tumor was histologically diagnosed as myofibroma, and we thus performed a marginal excision. The histopathological examination of the resected specimen revealed that the tumor was covered by a thin fibrous capsule, with a proliferation of enlarged vessels on the tumor surface (Figure 2A). Spindle cells with short spindle-shaped nuclei proliferated densely within the tumor, which contained blood vessels of various sizes (Figure 2B). The stroma was mildly edematous with lymphocytic infiltration (Figure 2C).

The immunohistochemical findings showed partial positivity for vimentin, alpha-smooth muscle actin (α-SMA), and progesterone receptor (PgR), and positivity for estrogen receptor (ER) (Table 1). Cyclin-dependent kinase 4 (CDK4) and murine double minute 2 (MDM2) showed nonspecific reactions, while CD34, desmin, S-100 protein, signal transducer and activator of transcription 6 (STAT6), and Retinoblastoma 1(RB1) were negative (Table 1). Based on these histological and immunohistochemical results, the tumor was finally diagnosed as CA. No local recurrence was observed 52 months postoperatively.

**Table 1:** Immunohistochemical findings of the three patients (Case 1, Case 2, Case 3) AR: androgen receptor; CDK4: cyclin-dependent kinase 4; EMA: epithelial membrane antigen; ER: estrogen receptor; MDM2: murine double minute 2; PgR: progesterone receptor; S-100: S-100 protein; STAT6: signal transducer and activator of transcription 6; α-SMA: alpha-smooth muscle actin; RB1: Retinoblastoma 1

Immunohistochemistry	Case 1	Case 2	Case 3
Positive	ER, PgR (partial), vimentin (partial), α-SMA (partial)	ER, PgR, vimentin, EMA, AR, CDK4, desmin	ER, PgR, EMA, AR
Negative	CDK4, S-100, desmin, STAT6, RB1	α-SMA, MDM2, CDK4, STAT6, RB1	α-SMA, S-100, desmin, STAT6, RB1

Case 2

A 33-year-old Japanese female patient with a history of glaucoma had noticed a gradually increasing subcutaneous mass in her right lower abdomen five years before her first visit to our hospital. She was referred to our department from the gynecology department because a soft tissue tumor was suspected. The physical examination identified a 15×16 cm elastic, soft mass in the patient's right lower abdomen. Although no tenderness was observed, pulsation was palpable on the caudal side of the mass.

**Figure 3:**
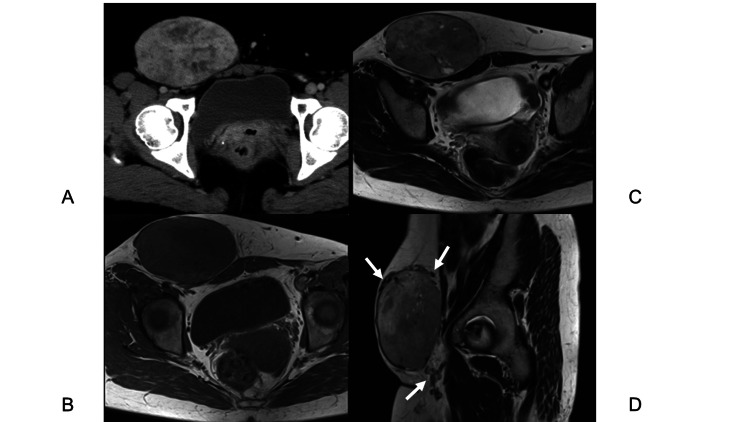
Contrast-enhanced CT and MRI of the pelvis (Case 2) A well-defined 11.8×12.2×6.9 cm tumor covered by a capsule and showing heterogeneous enhancement on contrast-enhanced CT (A: axial view), low- to iso-intensity on T1-weighted imaging (B: axial view) and heterogeneous intensity on T2-weighted imaging (C: axial view) is observed within the subcutaneous fat tissue adjacent to the rectus abdominis surface. Tortuous vascular structures (arrows) are proliferating around the tumor on T2-weighted imaging (D: sagittal view).

**Figure 4:**
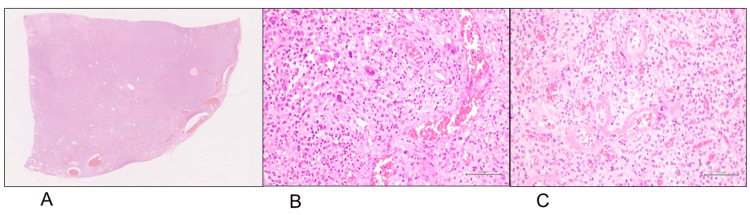
Histopathological findings, hematoxylin & eosin staining (Case 2) Abundant various-sized blood vessels were interspersed, with tumor cells having short spindle or round nuclei (A: low-power view; B: ×100). Hyalinization of blood vessels was noted, with inflammatory cell infiltration around the vessels (C: ×400).

MRI revealed an encapsulated 11.8×12.2×6.9 cm tumor within the subcutaneous soft tissue of the rectus abdominis surface. On MRI, the tumor exhibited low intensity on T1-WI (Figure 3A) and high intensity with spotty very high regions on T2-WI (Figure 3B), with tortuous vessels surrounding the tumor (Figure 3C). Because the biopsy specimen was pathologically diagnosed as CA, we performed a marginal excision. The histopathological examination of the resected specimen showed a fibrous capsule covering the tumor (Figure 4A), with abundantly interspersed vessels and a slightly dense proliferation of tumor cells with short spindle or round nuclei. Hyalinization of the vessel walls (Figure 4B) and infiltration of inflammatory cells around the vessels were observed (Figure 4C).

The immunohistochemical findings showed positivity for CD34, desmin, ER, PgR, vimentin, epithelial membrane antigen (EMA), and androgen receptor (AR), while α-SMA, MDM2, CDK4, STAT6, and RB1 were negative (Table 1), confirming the diagnosis of CA. No local recurrence was observed 30 months postoperatively.

Case 3

A 69-year-old Japanese male patient with a history of hypertension, cerebral infarction, gastric cancer, and right inguinal hernia had noticed a mass 15 years before his first visit to our hospital but left it untreated. A large mass extending from the patient's right inguinal region to the perineum was noted during his hospitalization for a cerebral infarction at the prior hospital. An incidental gastric cancer lesion was also detected and treated with a laparoscopic distal gastrectomy, during which a biopsy of the inguinal mass was performed. The results of the pathological examination suggested AA or CA, leading to his referral to our department.

**Figure 5:**
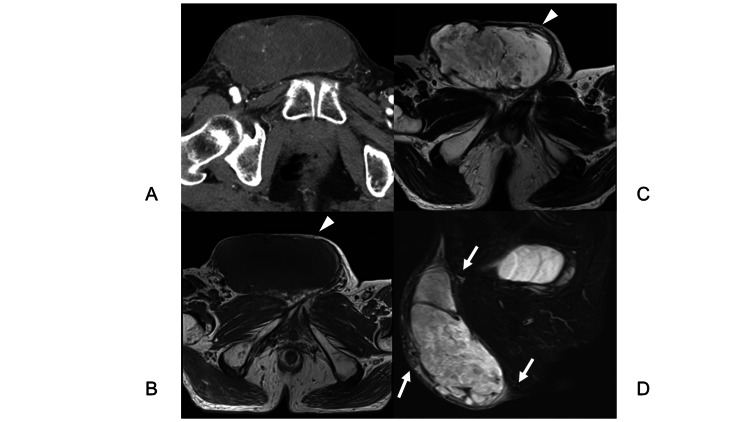
Contrast-enhanced CT and MRI of the pelvis (Case 3) Contrast-enhanced CT reveals an encapsulated subcutaneous 17.1×12.3×9-cm soft tissue tumor with numerous tumoral vessels (A: axial view). MRI reveals well-defined tumor (arrow heads) covered by a capsule is present within the subcutaneous fat tissue. The tumor exhibits iso-intensity on T1-weighted imaging (B: axial view), heterogenous high intensity on T2-weighted imaging (C: axial view) and enhancement on the distal side of the tumor on the gadolinium-enhanced T1-weighted fat-suppression image (D: sagittal view). Tortuous vascular structures (arrows) are proliferating around the tumor.

The initial examination identified a 20×16 cm subcutaneous elastic soft mass in the patient's inguinal region without redness, warmth, tenderness, or palpable pulsation. Contrast-enhanced CT revealed an encapsulated subcutaneous 17.1×12.3×9 cm soft tissue tumor with numerous tumoral vessels (Figure 5A). On MRI, the tumor exhibited iso-intensity on T1-WI (Figure 5B), heterogenous high intensity on T2-WI (Figure 5C) and short tau inversion recovery image (Figure 5D), and enhancement on the distal side of the tumor on the gadolinium-enhanced T1-weighted fat-suppression image. The tumor was adjacent to the patient's spermatic cord and penis, with surrounding tortuous vessels. Since a biopsy review confirmed the tumor as benign, we performed a marginal excision.

**Figure 6:**
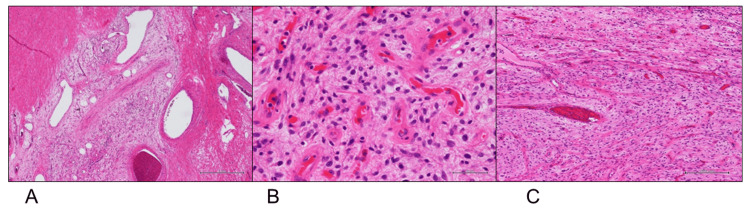
Histopathological results, hematoxylin and eosin staining (Case 3) Prominent vascular inflow is observed at the tumor periphery (A: ×12.5), with tumor cells having round-to-oval nuclei (B: ×100). There is an abundant interspersion of various-sized blood vessels, and variations in cellular density are observed (C: ×40).

The histopathological examination revealed a sparse to slightly dense proliferation of tumor cells with round-to-oval nuclei, with notable vascular inflow at the tumor periphery (Figure 6A, 6B). Abundant interspersed vessels of varying sizes were also observed within the tumor (Figure 6C). The immunohistochemical findings included positivity for ER, PgR, EMA, and AR, while α-SMA, S-100 protein, desmin, STAT6, and RB1 were negative, confirming the diagnosis of CA (Table 1). Although there was no local recurrence, the patient died of ischemic heart disease approximately one year after the surgery.

## Discussion

The preoperative imaging diagnosis is sometimes difficult since CA has many pathological and imaging findings similar to those of AMF and AA [2]. The MRI findings of CA depend on the amount of spindle cells, myxoid and collagenous stroma, and fat within the tumor. CA shows a well-defined border and typically iso-intensity on T1-WI, heterogeneous high intensity on T2-WI, and heterogeneous enhancement with gadolinium on T1-weighted fat suppression [3,4]. Additionally, the extent of tissue cellularity and increased cellularity are associated with restricted diffusion and reduced ADC, as in this case [3]. Pathologically, CA is a boundary-clear tumor characterized by short spindle-shaped cells and the proliferation of various-sized blood vessels [2,4,5], often accompanied by hyalinization of the vessels [6,7]. Immunohistochemistry for CAs has shown approximately 50-60% positivity for CD34, ER, and PgR, approximately 20% positivity for α-SMA, rare positivity for desmin [6-8], and complete negativity for RB1 (Table 2) [8,9].

**Table 2:** The clinical, imaging, and histopathological features of CA, AMF, and AA CE T1/FS: contrast-enhanced T1-weighted fat suppression; ER: estrogen receptor; PgR: progesterone receptor; α-SMA: alpha-smooth muscle actin; RB1: Retinoblastoma 1; AR: androgen receptor; CDK4: cyclin-dependent kinase 4; S-100: S-100 protein; MDM2: murine double minute 2

Features	Cellular angiofibroma (CA)	Angiomyofibroblastoma (AMF)	Aggressive angiomyxoma (AA)
Clinical	Slow-growing painless mass, roughly equally affecting males and female patients, with a peak incidence in the fifth decade of life in females and the seventh decade in males. Size: varies from 0.6 to 25 cm	Slow-growing painless circumscribed mass, arising predominantly in premenopausal female patients. Size: <5 cm (most cases), > 10 cm (rare)	Slow-growing, deep-seated painless mass, arising commonly in female patients in their fourth to fifth decades of life Size: >10 cm
Image (MRI)	Well-defined border tumor, T1-WI: iso intensity, T2-WI: heterogeneous high-intensity, CE T1/FS: heterogeneous enhancement	Well-circumscribed but not encapsulated tumor, T1-WI: iso intensity , T2-WI: high-intensity, CE T1/FS: uniform enhancement	Well-defined tumor, but often with unclear boundaries T1-WI: iso intensity, T2-WI: low intensity within the high-intensity tumor (swirl sign)
Histopathology	Uniform short spindle-shaped tumor cells in an oedematous to fibrous stroma and numerous various-sized blood vessels with hyalinization	Round to spindle-shaped tumor cells in abundant loose, oedematous stroma with small- to medium-sized vessels	Stellate or short spindle-shaped tumor cells with round-to-oval nuclei in mucinous stroma, and median- to large-sized vessels evenly dispersing throughout the tumor
Immunohistochemistry	Positive: ER, PgR (50-60%), α-SMA (20%), CD34, desmin (rarely) Negative: RB1	Positive: ER, PgR, desmin, vimentin, α-SMA (focal), Negative: CD34	Positive: ER, PgR, desmin, α-SMA, CD34, AR (Male), HMGA2, CDK4 Negative: S-100, MDM2

AMF is a painless benign tumor that grows over weeks and has a very low recurrence rate; marginal excision is its standard treatment [9]. MRI examinations of AMFs have shown iso-intensity on T1-WI, high intensity on T2-WI, and uniform enhancement on gadolinium-enhanced T1-weighed fat-suppression images [10]. Pathologically, AMF exhibits cellular density variations, with round and spindle cells around small- to medium-sized vessels [6,11] and edematous stroma [10]. Immunohistochemistry consistently shows positivity for ER, PgR, and desmin, with CD34 often negative (Table 2) [8].

AA is a well-defined tumor typically identified in the pelvis and perineum, characterized by local infiltration and frequent recurrence without distant metastasis [6]. Since AAs often present with unclear boundaries and because incomplete excision has led to local recurrence in 36-72% of cases, complete excision is desirable [6,12]. MRI examinations of AAs have shown iso-intensity on T1-WI and high intensity on T2-WI [10], with a swirling and layering pattern of low intensity within the high-intensity tumor on T2-WI [10,12].

Pathologically, AAs have low-to-moderate cellular density, with stellate or short spindle cells with round-to-oval nuclei infiltrating a mucinous stroma [6]. Immunohistochemistry shows positivity for desmin, α-SMA, ER, and PgR, with S-100 protein being negative [9,13]; male patient cases have also exhibited positivity for PgR and AR (Table 2) [13].

There are scattered reports of the presence or absence of blood vessels within the tumor, their size, and fatty infiltration as differential points on MRI [2,3,14]. Still, our search of the relevant literature revealed no mention of tortuous vessels around the tumor, which could help differentiate AA or AMF and CA in the preoperative imaging diagnosis.

All three patients in the current report had well-defined masses but the tumors were slightly larger than previously reported (ranging from 4-20 cm). MRI showed low to iso-intensity on T1-WI and high on T2-WI, and contrast-enhanced MRI showed enhancement on three tumors. The pathology showed spindle cells and blood vessels of various sizes, and hyalinization was also seen. Immunohistochemistry was positive for ER and PgR in all three cases and negative for RB1. Desmin was positive in only one of the three cases, mainly consistent with previous reports.

The development of peritumoral vascularity in CA is thought to be due to the structural characteristics of blood vessels and the histological characteristics of the tumor. The diameter of AMF is often smaller than that in CA. Small blood vessels with thin walls are observed in the edematous stroma of AMF. Medium to large blood vessels are scattered in AA, the vascular walls are hyalinized, and concentric clusters of collagen fibers may be seen around the blood vessels. However, because the stroma is mucinous and highly infiltrative [9,13], blood vessels are more likely to be distributed within the tumor than around the tumor. CA contains many small to medium-sized blood vessels with thick vascular walls [6]. In addition, because CA has a stroma with a high collagen content, it is thought that it limits the distribution of blood vessels within the tumor and instead promotes angiogenesis around the tumor [6-9,15]. The tortuous vessels have no specific name, but these blood vessels are thought to be connected to the femoral and the great saphenous veins. In the cases described in this report, the vascular network is believed to have developed as the tumor grew.

## Conclusions

CA, AA, and AMF have nonspecific imaging findings and can be difficult to differentiate preoperatively, even with a pathological examination. Our experience with the present three patients suggests that identifying significant vascular proliferation around the tumor can help us diagnose CA when differentiating soft tissue tumors around the perineal region from AMF and AA.
